# Rotating Machinery Fault Diagnosis Method by Combining Time-Frequency Domain Features and CNN Knowledge Transfer

**DOI:** 10.3390/s21248168

**Published:** 2021-12-07

**Authors:** Lihao Ye, Xue Ma, Chenglin Wen

**Affiliations:** 1 School of Automation, Hangzhou Dianzi University, Hangzhou 310018, China; yelihao@hdu.edu.cn (L.Y.); xuema@hdu.edu.cn (X.M.); 2 School of Automation, Guangdong University of Petrochemical Technology, Maoming 525000, China

**Keywords:** fault diagnosis, transfer learning, knowledge transferring, model transferring, deep CNN

## Abstract

Aiming at the problem of fault diagnosis when there are only a few labeled samples in the large amount of data collected during the operation of rotating machinery, this paper proposes a fault diagnosis method based on knowledge transfer in deep learning. First, we describe the data collected during the operation as a two-dimensional image with both time and frequency-domain characteristics. Second, we transform the trained source domain model into a shallow model suitable for small samples in the target domain, and we train the shallow model with small samples with labels. Third, we input a large number of unlabeled samples into the shallow model, and the output result of the system is regarded as the label of the input sample. Fourth, we combine the original data and the data annotated by the shallow model to train the new deep CNN fault diagnosis model so as to realize the migration of knowledge from the expert system to the deep CNN. The newly built deep CNN model is used for the online fault diagnosis of rotating machinery. The FFCNN-SVM shallow model tagger method proposed in this paper compares the fault diagnosis results with other transfer learning methods at this stage, and its correct rate has been greatly improved. This method provides new ideas for future fault diagnosis under small samples.

## 1. Introduction

In industrial production, any faults of a rotating machine may cause huge economic losses [1,2,3]. Therefore, fault detection and fault diagnosis are essential to the operational safety of modern manufacturing systems [4,5]. Due to the complexity of fault types, the high cost of manual annotation samples, and the lack of fault samples, the problem of fault diagnosis under small samples is still a research field that needs continuous optimization, continuous update, and progress [6,7].

Fault signals can generally be divided into the following three categories according to the input signal domain: time-domain [1,2,8], frequency-domain [9], and time-frequency domain [5,10,11,12]. In addition, fault diagnosis methods can be divided into methods based on shallow models and methods based on deep learning according to the difference of classifier models. The shallow model-based methods include Support Vector Machine (SVM) [13,14,15], Boosting [16,17], and Extreme Learning Machine (ELM) [18,19,20]. Deep learning-based methods require massive amounts of data to build multi-layer deep neural networks, such as Deep Belief Nets (DBN) [21,22] and Convolutional Neural Network (CNN) [20,23,24]. However, fault diagnosis methods based on shallow models face the problem of weak generalization ability, and fault diagnosis methods based on deep learning cannot meet the training needs of deep learning in a small sample environment [25,26,27,28].

Therefore, many scholars are committed to small sample learning work [29,30]. For the case where there is only a small number of labeled samples during the operation of the rotating machine, some scholars have proposed a method of constructing a feature pool. Zhang et al. proposed for the first time that by passing the original fault signal through the STFT, which can convert time domain information into time-frequency domain information, different special pools can be established based on the time-frequency domain information, and then, the features in the feature pool can be used to train the SVM for fault diagnosis tasks [12]. Some scholars have also proposed methods based on generative adversarial networks to face the problem of rotating machinery failures under small samples. Ali-Gombe et al. proposed the Few-shot Classifier Generative Adversarial Network as a small sample classification method. The images generated by their method can be divided into multiple pseudo-types or true-types. The key innovation of their adversarial approach is to allow the use of multiple pseudo-classes with semi-supervised deep learning for fine-grained classification. They achieved the purpose of small sample fault diagnosis by expanding the small sample [31]. The above two methods have good results in a small sample, but the method of Zhang et al. requires a lot of prior knowledge, and the generalization ability of the Ali-Gombe method is not strong enough.

On this basis, many scholars have proposed methods based on transfer learning to solve the problem of fault diagnosis under small samples. Snell et al. learn a metric space through a prototype network, in which classification can be performed by calculating the distance to the prototype representation of each class [32]. Huang et al. proposed a method of local migration and knowledge migration. They used the local migration of the model to achieve the final small sample fault diagnosis [33]. Although this method is more accurate than the previous method for fault diagnosis, its model migration part has too many training parameters, which is more costly in actual engineering. In some cases, this fault diagnosis accuracy rate and the method of migrating the entire model did not improve significantly.

Inspired by this, this article proposes the rotating machinery fault diagnosis method by combining time-frequency domain features and CNN knowledge transfer for the problem of only a small amount of data with expert annotations in the large amount of data collected during the operation of rotating machinery. First, we propose the FFCNN-SVM (Feature Fusion Convolutional Neural Network-Support Vector Machines) method to assign pseudo-labels to a large number of unlabeled samples. We can transform the source domain model by adding a new convolution layer and pool layer and retrain it so that it can extract the main features of the target domain failure under the condition of small sample training. Then, the SVM classifier that can be trained under small samples and has good generalization ability is connected to the last pooling layer of the model. The FFCNN-SVM method can have a good fault diagnosis effect with only a small number of training samples. However, the generalization ability of a single FFCNN-SVM model is not strong enough. So, we extend this work and proposed a new deep learning method that uses FFCNN-SVM for the annotation work of unannotated samples. The final model not only retains the expert knowledge transferred from the shallow model FFCNN-SVM but also has the characteristics of strong generalization ability of CNN. On the basis of the annotation content of these unannotated samples, we combine them with the original scarce sample set of the target domain to form an Augmented Training Set (ATS). The Augmented Training Set can be used to train a new deep CNN. Finally, we use this CNN to perform the online fault diagnosis of rotating machinery

The main contribution of this paper is that it is the first to propose a label data generation method based on mixed features by combining time-frequency domain features and CNN knowledge transfer. The specific contributions are as follows. (1) We combine the time-frequency domain feature method to make the task of labeling more accurate. (2) The method FFCNN-SVM of transformation and transfer is more suitable for small sample target domain situations. (3) The new CNN is designed to better fit to the target domain. (4) Compared with mainstream methods, it shows that this method has stronger knowledge transfer ability.

The main contents of the rest of this article are as follows: Section 2 introduces related work, including STFT, CNN, and an overview of transfer learning. The third section introduces the construction of the shallow model of the target domain and the rotating machinery fault diagnosis method that combines time-frequency domain features and CNN knowledge transfer. In Section 4, we show the experimental results of a rotating machinery fault diagnosis method that combines time-frequency domain features and CNN knowledge transfer. In Section 5, we summarize this article and propose several potential research directions.

## 2. Related Work

### 2.1. Short-Time Fourier Transform

STFT plays a vital role in studying time-varying and non-stationary signals. As a time-frequency analysis method, it plays a vital role in signal analysis. Through this transformation, a one-dimensional time series signal can be transformed into a two-dimensional matrix containing both time domain and frequency domain information. The basic idea of STFT is to intercept the time-domain signal with a fixed-length window function and use Fourier transform to analyze the intercepted time-domain signal to obtain the local frequency spectrum in a very small time period near time. The window function is shifted continuously on the entire time axis to finally obtain a partial frequency spectrum set. The mathematical expression is as follows:

(1) $$F_{i}(m,n)=\sum_{a=0}^{N-1}s_{i}(a)\cdot g^{*}(n-a)\cdot e^{-j\frac{2\pi }{N}\cdot ka}$$

where $s_{i}(a)$ is the channel wave signal of the *i*-th sample, $g^{*}(\cdot )$ is the window function, and $F_{i}(m,n)$ is the result of STFT, which describes the characteristics of the frequency amplitude change over time.

Although CNN does not require the form of input data, even the original vibration signal can also be used as the input of CNN. However, adopting certain methods to preprocess the input data can improve the diagnosis accuracy to a certain extent. The research surface in the field of speech recognition: the recognition effect of using the speech spectrogram (the result of STFT transformation) as the input of CNN is better than the Mel-Frequency Cepstrum Coefficents (MFCC) [34,35]. Inspired by this, we first transform the one-dimensional fault signal into the time-frequency domain through STFT to obtain the time-frequency characteristics of the fault signal. The time-frequency diagram of a signal describes the relationship between frequency and time.

### 2.2. Convolutional Neural Network

**Figure 1:**
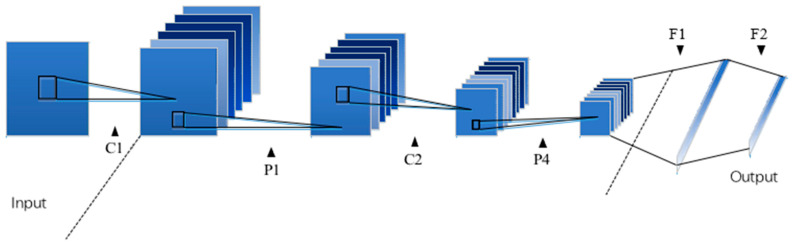
CNN network structure.

Convolutional Neural Network (CNN) is one of the representative network structures in the field of deep learning [36]. Figure 1 shows the structure of a classic CNN model.

Figure 1 describes a more common CNN model, which will be introduced as an example below. The convolutional layer C1-P1-C2-P2 is the core part of CNN. Its role is to extract image features, and the subsequent F1-F2 is the fully connected part, and its main task is to perform fault diagnosis tasks. First, the original training picture is input to the convolutional layer of the CNN neural network. In the convolutional layer C, through several convolution kernels of the same size, the convolution operation result of this picture through several convolution kernels can be output. In Figure 1, there is an activation function operation between C and P. The activation function enables the convolution result to fit the nonlinear system, and the P after this represents the maximum pooling layer. The maximum pooling layer makes it possible to effectively compress the image and reduce the number of training parameters by selecting the maximum value of the area in a certain area as the representative of the area. The FC layer in the figure means a fully connected layer, and there are weighted connections between the fully connected layers, and the full connection can learn these nonlinear combination characteristics in a simple way.

#### 2.2.1. Activation Function ReLU

The introduction of the activation function is to increase the nonlinearity of the neural network model. The introduction of the activation function can make the neural network approximate any nonlinear function, so that the neural network can be applied to more scenarios. If there is no activation function, each layer of the neural network is equivalent to the linear combination of the previous layer; then, the middle layer has no meaning.

Among them, the ReLU activation function is a widely used activation function, which is specifically expressed as:

(2) f(x)=max(0,x).

In deep learning, the biggest problem is the disappearance of gradients. With its linear and non-saturated form, the ReLU function can not only solve the problem of disappearing gradients in network direction error propagation but also speed up training. Therefore, the activation layer in this paper uses the ReLU function as the activation function.

#### 2.2.2. Stochastic Gradient Descent

The most commonly used optimization method for deep learning is Stochastic Gradient Descent (SGD). The advantages of stochastic gradient descent are mainly reflected in two aspects: high efficiency and fast execution speed. At the same time, compared with other popular optimization methods (e.g., Adam), it requires a large number of hyperparameters and is sensitive to feature transformation.

If a training sample set is given as $\left(x_{1},y_{1}\right)$,…, $\left(x_{n},y_{n}\right)$, $x_{i}\in R^{m}$, $y_{i}\in \{-1,1\}$, our goal is to learn a linear scoring function as $f(x)=\omega^{T}\cdot x+b$, where the model parameters are $\omega \in R^{m}$, b∈R. A common method for estimating model parameters is to minimize the regular training error:

(3) $$E(\omega ,b)=\frac{1}{n}\sum_{i=1}^{n}L(y_{i},f(x_{i}))+\alpha R(\omega ).$$

Here, L is the loss function, which will be introduced in the next section, R is the penalty term, and α is a non-negative hyperparameter. SGD is a commonly used optimization method. Compared with traditional gradient descent, SGD approximates the true gradient of E(ω,b), considering one training sample at a time. Update the parameters according to the following formula:

(4) $$\omega \leftarrow \omega -\eta (\alpha \frac{\partial R(\omega )}{\partial \omega }+\frac{\partial L(\omega^{T}x_{i}+b\cdot y_{i})}{\partial \omega }).$$

Here, η is the learning rate, which is used to control the search step length of the parameter space, which is either a constant or gradually becomes smaller. The learning rate for classification problems is usually defined by:

(5) $$\eta^{\left(t\right)}=\frac{1}{\alpha (t_{0}+t)}$$

where t is the time step. All optimization methods of convolutional neural networks in this article use the Stochastic Gradient Descent method to update network parameters.

#### 2.2.3. Cross-Entropy Loss

The loss function can be a good measure of the predictive ability of the machine learning model. It can provide an optimization direction of machine learning model; no single loss function can be applied to all problems. The choice of loss function depends on many factors, including whether there are outliers, the choice of machine learning algorithm, whether it is easy to find the derivative of the function, and the confidence of the predicted result.

All the loss functions used in the model training process in this paper adopt the cross-entropy loss function, which is defined on the basis of the probability distribution. This mathematical formula can be described as:

(6) $$Loss=-[y\log\hat{y}+(1-y)\log(1-\hat{y})].$$

### 2.3. Transfer Learning

The goal of transfer learning is to establish a reliable model based on the number of samples and laboratory data with sufficient label information to predict the actual engineering data of samples and insufficient labels under different distributions. Source domain data sets with sufficient health labels can be obtained in the laboratory as ${\left\{x_{i}^{S},y_{i}^{S}\right\}}_{i=i}^{n_{s}}$, in which $x_{i}^{S}\in X^{S}$ and its data distribution obeys the marginal probability distribution $P(X^{S})$, among which $y_{i}^{S}\in Y^{S}$ is the health status label of the sample. Using sufficient fault data and status labels in the source domain, a mapping function $f^{S}(●)$ from samples to labels can be established. Due to different equipment operating conditions and models, the probability distributions between different data sets are different, and the mapping function established on the source domain cannot be directly used for the fault classification of the target domain.

**Figure 2:**
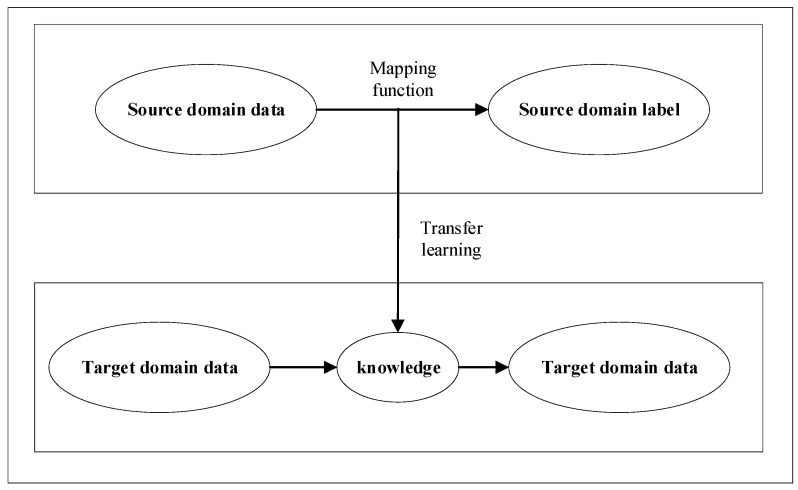
Schematic diagram of transfer learning principle.

Therefore, the task of transfer learning is shown in Figure 2. For a given target domain, with the help of the existing source domain and source task knowledge, a mapping function from target domain data to tags is established to complete the target task.

Transfer learning is an important idea of this article. Since there are fewer manually labeled samples in the target domain, we need to find a source domain and get a model of the target domain based on the transformation of the source domain model, so that even under the condition of scarce value samples, we can get a good model of the target domain to diagnose the fault.

## 3. The Proposed Method

The main content of this section (1) explains a new FFCNN-SVM method, which can diagnose faults well in the case of a small sample of the target domain; and (2) re-establishes a CNN that is more suitable for the target domain through the ATS.

### 3.1. FFCNN-SVM Model Transfer Method Based on Transfer Learning

The goal of this section is to describe a new method that can use the source domain model to transform the target domain model when the sample size in the target domain is small, and the fault diagnosis rate of this model is very high.

**Figure 3:**
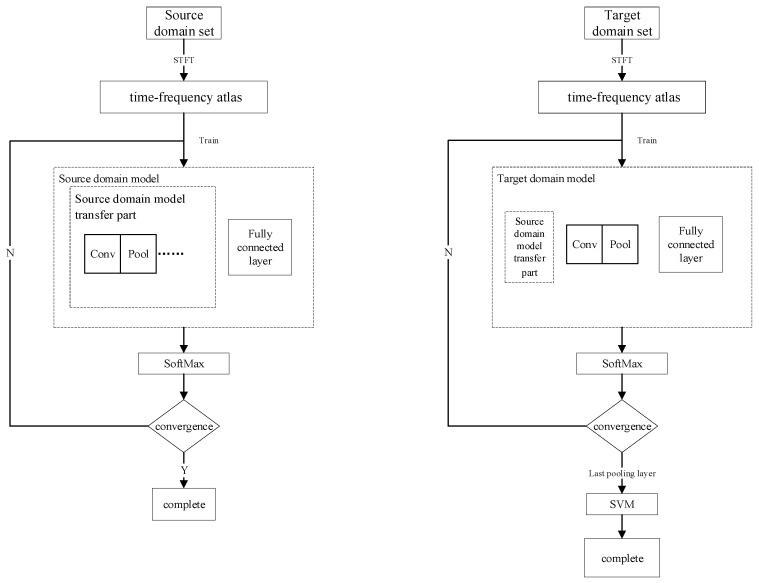
FFCNN-SVM model fault diagnosis flowchart.

We propose a new deep CNN method based on FFCNN-SVM based on transfer learning. This method is used for the fault diagnosis of rotating electric machines with a large number of unlabeled samples. The fault diagnosis method is shown in Figure 3. The specific process steps are as follows.

First, we convert the collected vibration signal $s_{i}(a)$ into a two-dimensional image structure time-frequency image F(m,n) through Short-Time Fourier Transform. STFT is performed on the different acquisition channels of each sample, and finally, we can get a sample with a three-dimensional structure, namely F(m,n,K).

After that, the source domain CNN model can be trained by using the source domain spectrum atlas. First, the atlas is convolved by the convolutional layer, and the role of the convolutional layer is to extract local area features; different convolution kernels are equivalent to different feature extractors.

For the lth layer as a convolutional layer, the input map of the input feature map of the (l−1)th layer is $x^{l-1}\in R^{m\times n\times K}$, the net input of the feature map of the lth layer is obtained by convolution calculation as $Z^{l}\in R^{m^{*}\times n^{*}\times P}$. The net input of the pth feature map of the lth layer is as follows:

(7) $$Z^{\left(l,p\right)}=W^{\left(l,p\right)}⊗X+b^{\left(l,p\right)}=\sum_{k=1}^{K}W^{\left(l,p,k\right)}⊗X^{\left(l-1,k\right)}+b^{\left(l,p\right)}$$

where $W^{\left(l,p,k\right)}$ and $b^{\left(l,p\right)}$ are the convolution kernel and the bias; there are a total of P×K convolution kernels and P bias in the lth layer.

On the basis of the convolutional layer, we also use the maximum pooling layer, which can effectively reduce the number of parameters. The maximum pooling layer is for a region $R_{m,n}^{k}$ to select the maximum activity value of all neurons in this region as the representation of this region. Do maximum pooling on the output of the convolutional layer of the lth layer, as follows:

(8) $$y_{m,n}^{\left(l,k\right)}=\underset{i\in R_{m,n}^{k}}{\max}x_{i},$$

After the feature extraction layer, there are fully connected layers and SoftMax classifiers. We use a large number of fault spectrograms after STFT to train the source domain model $M^{Source}$.

In order to achieve the final fault diagnosis in the target domain, we need to reconstruct the source domain model.

The model $M^{T1}$ is reformed from the source domain model $M^{Source}$. We added a new convolutional layer and pooling layer that need to be trained on the basis of the source domain model and froze the feature layer extracted from the source domain before. Then, we used the scarce manual sample spectrum atlas of the target domain to train the model.

After the $M^{T1}$ model training converges, the output of the last pooling layer is input to the SVM classifier. Then, the scarce manual samples of the target domain are input into the model to train the final SVM classifier, and the final fault diagnosis model can be obtained after the training is completed. We call this method the FFCNN-SVM method.

### 3.2. Knowledge Transfer from Shallow Model to Deep Learning Model

The goal of this section is to use ATS to train a CNN that is more fitted to the target domain.

**Figure 4:**
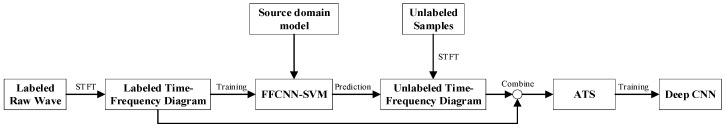
Block diagram of the process of transferring knowledge from a shallow model to a deep learning model.

Although the FFCNN-SVM method can reveal some inherent characteristics of the fault sample based on a small amount of data, when there is a large amount of data in the target domain that needs to be fault diagnosed, although the fault diagnosis rate is high, it may still produce a large number of fault errors. Therefore, this method can only be constructed as a shallow model. The label of the target domain predicted by the shallow model can be regarded as the knowledge of the target domain. We combine the label samples of the target domain with the label samples predicted by the shallow model to form the Augmented Training Set (ATS) and use this data set to train the final deep CNN model. From this process, we can obtain a CNN classifier that can reveal more fault features and a stronger diagnostic effect, thereby realizing the knowledge transfer from the shallow model FFCNN-SVM to deep CNN to get a better model. The specific flow chart is shown in Figure 4.

## 4. Experiments

In order to evaluate the effectiveness of the proposed method, this paper conducts experiments on two rotating machinery failure data sets. We explain that the content mainly has the following two parts: the model transfer method FFCNN-SVM, which evaluates the transfer of the source domain model to the target domain model, and the evaluation of the deep CNN model based on knowledge transfer.

### 4.1. Case One

(1) Data set description and description of some parameters

**Figure 5:**
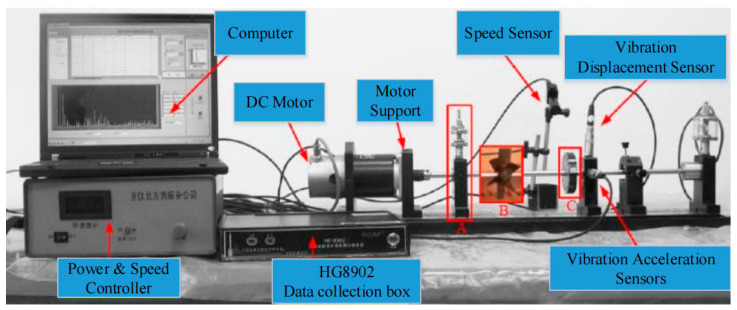
ZHS-2 type multifunctional motor test bench.

The rotating machinery fault diagnosis data sets we use are all from the ZHS-2 multifunctional motor platform shown in Figure 5. All signals are collected by the HG8902 data collection box in Figure 5. In this case, the data set has seven types of faults: Rotor Unbalance I, Rotor Unbalance III, Rotor Unbalance V, Rotor Unbalance VII, Pan Page Break, Pedestal Looseness, and the Normal condition. The four faults of rotor unbalance are simulated by installing different numbers of screws at the position shown in Figure 5C. The Pan Page Break fault is simulated by installing a page breaker on the drum at the position of Figure 5B. Pedestal Looseness is simulated by loosening the base bolts at the position of Figure 5A.

During this experiment, the acquisition time of each sample lasts for 8 s, and there were 8 sensors with different positions, each of which recorded 10,240 data points. A total of 300 samples were collected for each fault type and the normal conditions.

In addition, in order to illustrate the difference between the source domain and the target domain, we divided 2100 samples into two parts. Among them, the Pan Page Break, Pedestal Looseness, Rotor Unbalance I, and the Normal condition are combined, 1200 samples constitute the original data set of the source domain, and Rotor Unbalance III, Rotor Unbalance V, Rotor Unbalance VII, and normal state total 1200 samples constitute the original data set of the target domain. In the target domain, in order to illustrate the small sample status, the training sample capacity of the target domain is 2% of the total data set sample capacity of the target domain, a total of 24, and among these, 24 training samples and test samples are allocated according to 2:1. The remaining 1176 samples together constitute the target domain’s unlabeled sample set. The details are shown in Table 1.

Among them, the parameters of STFT are set as follows: For the window function, we choose a Hamming window with a length of 256, and its overlap size is 128. After STFT, the original signal of 10,240 sampling points can be turned into a two-dimensional spectrogram with a size of 32 × 128. There are eight sensors to collect waveform signals, so after STFT, the input size of the convolutional neural network is 32 × 128 × 8

(2) Model transfer method FFCNN-SVM based on feature fusion

**Figure 6:**
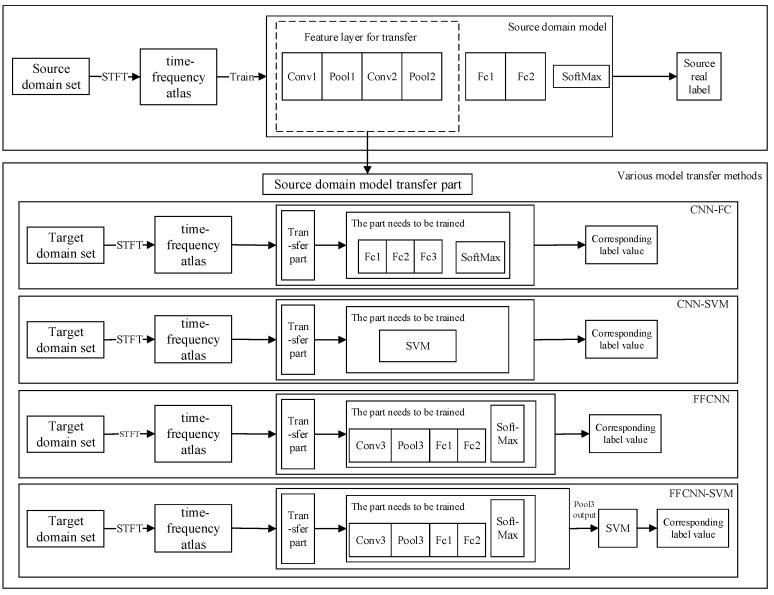
Flow chart of various model transfer methods.

There are many ways to transfer models from the source domain to the target domain. The following will introduce the CNN-FC model transfer method, CNN-SVM model transfer method, FFCNN model transfer method, and FFCNN-SVM model transfer method. The flowchart of various model transfer methods is shown in Figure 6.

In the simulation process of the FFCNN-SVM experiment, two types of CNN models with different levels of complexity were formed for the preliminary models of the source domain and the target domain. The specific network structure is shown in Table 2.

Each of the mentioned CNN models has undergone 10,000 iterations with a batch size of 256, and the initial learning rate is set as 0.01. In the iterative process, the learning rate is reduced by 90% every 2500 iterations. The momentum and the decay parameter are set to 0.9 and 5 × 10^−6^.

After the preliminary model of the target domain is trained, the convolutional layer and pooling layer of the target domain also extract the sample features of the target domain based on the source domain features. After the initial model training converges, connect its final maximum pooling layer to the SVM classifier. Re-input the target domain training set to the model to train the SVM.

In order to compare the FFCNN-SVM method with other model transfer methods and verify in different methods, we compare with the traditional SVM training method used in the target domain, as well as the CNN-FC method, FFCNN, and CNN-SVM method. The schematic diagram of the above several transfer learning methods is shown in Figure 6. The correct rate of the target domain test set is used as an index to evaluate the ability of model transfer, which is defined as follows:

(9) $$Accuacy=\frac{\sum_{i=1}^{C}C_{ii}}{\sum_{i=1}^{C}\sum_{j=1}^{C}C_{ij}}.$$

Here, $C_{ij}$ is the number of samples that belong to the i−th category and are predicted to the j−th category. C is the number of categories. Table 3 shows the classification accuracy of different methods in this experiment.

It can be seen from Table 3 that the shallow model using FFCNN-SVM can make a very high fault diagnosis accuracy rate when there is only a small number of training data in the target domain. Therefore, this method is used as a shallow model of the target domain for subsequent marking of a large number of unlabeled samples.

(3) Knowledge-transferring for deep CNN

After pseudo-labeling a large number of unlabeled samples by the FFCNN-SVM model, we combine this part of the sample with the training samples of the target domain to form an ATS. After having the ATS, we already have the conditions to establish our own deep learning framework for the target domain. On this basis, we constructed a CNN, as shown in Table 4.

Then, use ATS as the training set of the new CNN model. Realize the knowledge transfer of the shallow model. After the new CNN converges, its accuracy on the target domain test set is shown in Table 5. We can see that the accuracy of the CNN trained with ATS on the same test set is much higher than that of the CNN trained with the original training set of the target domain. This means that the CNN trained by ATS has learned more discriminative characteristics of the fault.

This experimental conclusion shows that our newly constructed CNN has fully adapted to the target domain and can make good fault diagnosis and modification.

### 4.2. Case Two

(1) Data set description and description of some parameters

**Figure 7:**
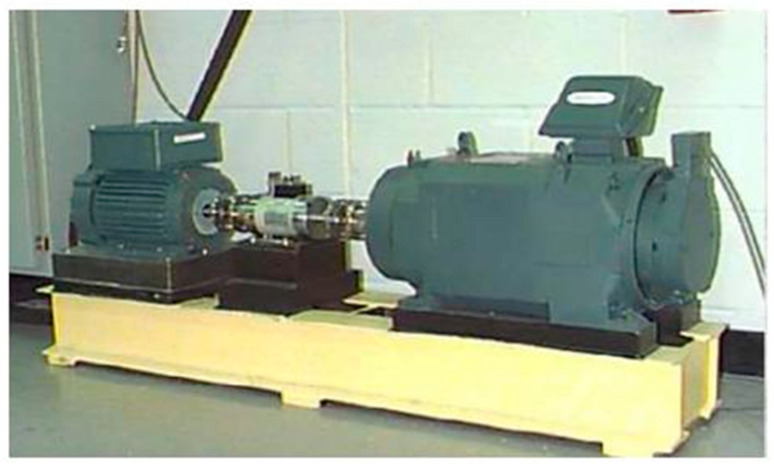
The test platform of CRWU.

The roller bearing data sets we use are all from the public datasets of Case Western Reserve University (CRWU) [37,38]. Figure 7 shows the test platform of CRWU. In this case, we used the data with a motor load of 1 horsepower and eight types of faults as our data set: Ball Defect I, Ball Defect II, Ball Defect III, Inner Race Defect I, Inner Race Defect II, Inner Race Defect III, Outer Race Defect I, and Outer Race Defect II. During this experiment, there were three sensors with different positions, each of which recorded 400 data points. A total of 300 samples were collected for each fault type and the normal conditions, and a total of 2400 samples are collected.

To illustrate the transfer effect of the source domain to the target domain, the source domain data set is Ball Defect I, Ball Defect II, Inner Race Defect I, Inner Race Defect II; the target domain data set is Ball Defect III, Inner Race Defect III, Outer Race Defect I, Outer Race Defect II. The details are shown in Table 6.

Among them, the parameters of STFT are set as follows: For the window function, we choose a Hamming window with a length of 16, and its overlap size is 8. After STFT, the original signal of 40 sampling points can be turned into a two-dimensional spectrogram with a size of 8 × 64. There are three sensors to collect waveform signals, so after STFT, the input size of the convolutional neural network is 8 × 64 × 3.

(2) Model transfer method FFCNN-SVM based on feature fusion

In the process of model transfer from the source domain to the target domain, various transfer methods are shown in Figure 6 of Case 1. The model structure of the source domain model and the target domain preliminary model is shown in Table 7.

Table 8 shows the classification accuracy of different methods in this experiment.

It can be seen from Table 8 that the shallow model using FFCNN-SVM can make a very high fault diagnosis accuracy rate when there is only a small number of training data in the target domain. Therefore, this method is used as a shallow model of the target domain for subsequent marking of a large number of unlabeled samples in the target domain.

(3) Knowledge-transferring for deep CNN

Through the FFCNN-SVM model, a large number of unlabeled data sets in the target domain can be labeled. Then, this part of the data set with pseudo-labels is combined with the training set to form an ATS. After having the ATS, we already have the conditions to establish our own deep learning frame-work for the target domain. On this basis, we build a deep CNN structure to get the final high-accuracy fault diagnosis model. The results of the fault diagnosis experiment on the test tags can be seen in Table 9.

This experimental conclusion shows that our newly constructed CNN has fully adapted to the target domain and can make good fault diagnosis and modification.

## 5. Conclusions and Discussions

This paper mainly introduces a rotating machinery fault diagnosis method combining time-frequency domain features and CNN knowledge transfer. This method uses the knowledge of the FFCNN-SVM shallow model transferred from the source domain to transfer to the final deep CNN model, and it successfully solves the problem of deep learning in the case of small samples. The method has the following three parts. (1) First, there is the training of the source domain model. (2) Second, there is the training of the shallow model FFCNN-SVM of the target domain and the use of the shallow model to attach pseudo-labels to unlabeled samples in the target domain. Finally, the target domain source labeled samples and pseudo-labeled samples are combined to form an Augmented Training Set (ATS). (3) Lastly, the deep CNN model is trained through the ATS to realize knowledge transfer. The experimental results on two fault diagnosis data sets show that the method has good performance and can be used in future work. At the same time, the application of this method is not limited to traditional fault diagnosis. This method can also be applied to other deep learning and artificial intelligence fields. In the foreseeable future work, such as the task of training neural network models with small samples against the generation network, the idea of this method can also be used.

## Figures and Tables

**Table 1:** Source/Target domain data classification table.

Domain	Data Set	Number of Data
Source domain	Training set	1200
Target domain	Training set	16
Target domain	Unlabeled set	1176
Target domain	Test set	8

**Table 2:** Source domain model and target domain preliminary model network structure.

Type	Source Domain Model					Target Domain Model				
	Input Size	Filter	Number	Padding	Stride	Input Size	Filter	Number	Padding	Stride
Conv1	32 × 128 × 8	(3,3)	32	(1,1)	(1,1)	32 × 128 × 8	(3,3)	32	(1,1)	(1,1)
Pool1	32 × 128 × 32	(4,8)	-	(4,8)	-	32 × 128 × 32	(4,8)	-	(4,8)	-
Conv2	8 × 16 × 32	(3,3)	32	(1,1)	(1,1)	8 × 16 × 32	(3,3)	32	(1,1)	(1,1)
Pool2	8 × 16 × 32	(4,8)	-	(4,8)	-	8 × 16 × 32	(4,8)	-	(4,8)	-
Conv3	-	-	-	-	-	2 × 2 × 32	(3,3)	32	(1,1)	(1,1)
Pool3	-	-	-	-	-	2 × 2 × 32	(2,2)	-	(1,1)	-
Fc1	2 × 2 × 32	-	64	-	-	1 × 1 × 32	-	16	-	-
Fc2	64	-	4	-	-	16	-	4	-	-

**Table 3:** Classification accuracy of different methods for unlabeled sample sets.

Classification Method	Unlabeled Set Accuracy	Parameter Amount
SVM	0.392	-
CNN-FC	0.71003	18,595
CNN-SVM	0.827	-
FFCNN	0.7253	9827
**FFCNN-SVM**	**0.956**	**-**

**Table 4:** Final deep CNN structure diagram.

Type	Input Size	Filter	Number	Padding	Stride
Conv1	32 × 128 × 8	(3,3)	32	(1,1)	(1,1)
Pool1	32 × 128 × 32	(4,8)		(4,8)	-
Conv2	8 × 16 × 32	(3,3)	32	(1,1)	(1,1)
Pool2	8 × 16 × 32	(4,8)	-	(4,8)	-
Fc1	2 × 2 × 32		64		
Fc2	64		4		

**Table 5:** Accuracy of the models.

	Target Domain Test Set Accuracy
CNN(Train)	1
CNN(Test)	0.75
CNN-ATS(Train)	1
**CNN-ATS(Test)**	**1**

**Table 6:** Source/Target domain data classification table.

Domain	Data Set	Number of Data
Source domain	Training set	1200
Target domain	Training set	16
Target domain	Unlabeled set	1176
Target domain	Test set	8

**Table 7:** Source domain model and target domain preliminary model network structure.

Type	Source Domain Model					Target Domain Model				
	Input Size	Filter	Number	Padding	Stride	Input Size	Filter	Number	Padding	Stride
Conv1	8 × 64 × 3	(3,3)	32	(1,1)	(1,1)	8 × 64 × 3	(3,3)	32	(1,1)	(1,1)
Pool1	8 × 64 × 32	(2,4)	-	(2,4)	-	8 × 64 × 32	(2,4)	-	(2,4)	-
Conv2	4 × 16 × 32	(3,3)	32	(1,1)	(1,1)	4 × 16 × 32	(3,3)	32	(1,1)	(1,1)
Pool2	4 × 16 × 32	(2,4)	-	(2,4)	-	4 × 16 × 32	(2,4)	-	(2,4)	-
Conv3	-	-	-	-	-	2 × 4 × 32	(3,3)	32	(1,1)	(1,1)
Pool3	-	-	-	-	-	2 × 4 × 32	(2,4)	-	(1,1)	-
Fc1	2 × 4 × 32	-	64	-	-	1 × 1 × 32	-	16	-	-
Fc2	64	-	4	-	-	16	-	4	-	-

**Table 8:** Classification accuracy of different methods for unlabeled sample sets.

Classification Method	Unlabeled Set Accuracy	Parameter Amount
SVM	0.5773	-
CNN-FC	0.7057	17,556
CNN-SVM	0.9846	-
FFCNN	0.72193	11,380
**FFCNN-SVM**	**0.99489**	**-**

**Table 9:** Accuracy of the models.

	Target Domain Test Set Accuracy
CNN (Train)	1
CNN (Test)	0.75
CNN-ATS (Train)	1
**CNN-ATS (Test)**	**1**
